# Prior Antithrombotic Therapy Is Associated With Cerebral Microbleeds in Ischemic Stroke Patients With Atrial Fibrillation and/or Rheumatic Heart Disease

**DOI:** 10.3389/fneur.2018.01184

**Published:** 2019-01-11

**Authors:** Yajun Cheng, Junfeng Liu, Shuting Zhang, Jie Li, Chenchen Wei, Deren Wang, Jing Lin, Yanan Wang, Bo Wu, Shihong Zhang, Ming Liu

**Affiliations:** ^1^Department of Neurology, Center of Cerebrovascular Disease, West China Hospital, Sichuan University, Chengdu, China; ^2^Department of Neurology, People's Hospital of Deyang City, Deyang, China

**Keywords:** antithrombotic therapy, cerebral microbleeds, ischemic stroke, atrial fibrillation, rheumatic heart disease

## Abstract

**Background and purpose:** Cerebral microbleeds (CMBs) could contribute to an increased risk of intracerebral hemorrhage in patients with antithrombotic therapy (antiplatelets or anticoagulants). Antithrombotic agents are commonly prescribed to the patients with atrial fibrillation (AF) and/or rheumatic heart disease (RHD) for preventing ischemic stroke. However, the impact of antithrombotic therapy on CMBs remained controversial. We aimed to explore the association between the prevalence of CMBs and prior antithrombotic therapy in ischemic stroke patients with AF and/or RHD.

**Materials and Methods:** Ischemic stroke patients with AF and/or RHD within 7 days of onset from two hospitals were enrolled. Clinical information, prior use of antiplatelets or anticoagulation, presence and location of CMBs on susceptibility weighted imaging were recorded. We investigated the association of antithrombotic use with the presence or location of CMBs using multivariable logistic regression.

**Results:** A total of 160 patients (68 males; median age, 71 years) were included. CMBs were observed in 90 (56.3%) patients, of whom 37 were with strictly lobar CMBs and 53 were with deep or infratentorial CMBs. There was a significant difference in antiplatelet use between patients with and without CMBs (33.3 vs. 11.4%, *P* = 0.001), but not found in anticoagulants. Prior use of antiplatelets was independently associated with the presence of CMBs (OR 3.075, 95% CI 1.175–8.045, *P* = 0.022) and especially strictly lobar CMBs (OR 2.635, 95% CI 1.050–6.612, *P* = 0.039) in multivariate analysis.

**Conclusions:** The present study suggests that CMBs are common in ischemic stroke patients with AF and/or RHD and prior antiplatelet use may relate to the presence of CMBs predominantly in the strictly lobar region. Whether anticoagulants could cause CMBs need to be determined in future longitudinal studies.

## Introduction

Ischemic stroke patients attributable to atrial fibrillation (AF) and rheumatic heart disease (RHD) are increasing in China, with a high rate of death and recurrence (1, 2). Although antithrombotic therapy (antiplatelets or oral anticoagulants) has been the mainstay for primary and secondary prevention of ischemic stroke in patients with AF and/or RHD, its use in clinical practice is suboptimal due to the feared risk of catastrophic intracerebral hemorrhage (ICH) (3). Therefore, risk stratification strategy is needed to balance the risk of ICH vs. ischemic stroke and guide the safe use of antithrombotic agents in Chinese stroke patients with AF/RHD, who have a heightened risk of ICH (4).

Cerebral microbleeds (CMBs) are key neuroimaging markers of hemorrhagic small vessel disease in the brain, which can be detected on bleeding sensitive magnetic resonance imaging (MRI) sequences such as gradient-echo T2^*^-weighted imaging (T2^*^-GRE) and susceptibility-weighted imaging (SWI) (5). Histopathologically, CMBs are perivascular hemosiderin deposits after small hemorrhages, indicative of bleeding-prone microangiopathy (6). They are commonly detected in healthy elderly and more prevalent in patients with ischemic or hemorrhagic strokes (7–9). The presence of CMBs has been reported to increase the risk of ICH up to 8-fold in a pooled cohort of ischemic stroke or transient ischemic attack (TIA) (10). This is more clinically relevant in AF/RHD patients with anticoagulation needs (11). Prior studies showed that CMBs appear to be more prevalent in patients with AF (12) and ≥5 CMBs powerfully predict future ICH risk in ischemic stroke patients with AF taking oral anticoagulants (13). Consequently, a challenging clinical dilemma emerged as whether antithrombotic decisions would be influenced by the presence of CMBs in patients with AF and/or RHD.

Since CMBs may represent hemorrhage-prone pathological states, the impact of different antithrombotic therapy on the presence of CMBs has been of great concern. Some previous studies showed that antiplatelet or anticoagulant use was significantly associated with the presence of CMBs in ischemic stroke patients (12, 14). However, the results regarding this question remained controversial and uncertain (15, 16). In addition, few studies (12, 17) on this question focused on stroke patients with AF and/or RHD. Besides, most studies observed CMBs on T2^*^-GRE, not on SWI, though SWI has been proven to be more sensitive at detecting CMBs (18).

Hence, we aimed to investigate the relationship between prior antithrombotic therapy and prevalence of CMBs observed on SWI in ischemic stroke patients with AF and/or RHD. Furthermore, we analyzed this association in different locations of CMBs with the presumption that strictly lobar or deep/infratentorial CMBs may have different underlying pathogenesis and different bleeding risk (5).

## Materials and Methods

### Study Population

This research is part of the project, “Study on small vessel pathological mechanism of cerebral hemorrhage after cardioembolic stroke using SWI markers,” approved by the National Natural Science Foundation of China. Ischemic stroke patients with AF and/or RHD were consecutively registered after being admitted to the department of neurology in West China Hospital, Sichuan University (10/2013-09/2016) and People's Hospital of Deyang City (09/2014-07/2015). The study protocol was approved by the medical ethics committee in each hospital. Written informed consent was obtained from participants or their guardians.

Ischemic stroke was diagnosed according to World Health Organization criteria and confirmed by computed tomography scanning or magnetic resonance imaging. AF was diagnosed based on a history of persistent AF or paroxysmal AF, supported by previous electrocardiograms or by electrocardiography (24-h or not) on admission (1). RHD was defined according to criteria in the International Classification of Diseases (10th edition) and confirmed by echocardiography.

### Data Collection

Ischemic stroke patients with AF and/or RHD within 7 days of symptom onset who had completed MRI including fluid-attenuated inversion recovery and SWI were enrolled in our study. We used a standardized form to extract patient's information including age, gender, National Institutes of Health Stroke Scale (NIHSS) score on admission, medical history of hypertension, diabetes mellitus, hyperlipidemia, and stroke/TIA, renal impairment (based on medical history or on prospective measurement of estimated glomerular filtration <60 ml/min/1.73 m) (19), current smoking and alcohol consumption, coagulation function on admission including prothrombin time (PT), activated partial thromboplastin time (APTT), and international normalized ratio (INR). We also collected information about patient-reported use and type of antithrombotic drugs before admission.

### Imaging Protocol

The parameters of MRI remained the same during the study period: (1) West China Hospital, Sichuan University (3.0 Tesla Siemens Trio MR scanner); (2) People's Hospital of Deyang City (1.5 Tesla Philips Achieva MR scanner). Details of the parameters of fluid-attenuated inversion recovery and SWI sequence have been described in our previous study (20).

### Assessment of Cerebral Microbleeds and White Matter Hyperintensity

A CMB was defined as a homogeneous, round focal area, with a diameter <10 mm and very low signal intensity on SWI (21). The location of the CMBs was classified as strictly lobar CMBs (1 or more microbleeds distributed in lobar location strictly) and deep or infratentorial CMBs (at least 1 microbleed in a deep or infratentorial brain location with or without concomitant lobar CMBs). The white matter hyperintensity (WMH) was assessed based on Fazekas scale (22). Two neurologists blinded to clinical data assessed CMBs and WMH independently. In case of disagreement, a third neurologist was consulted, and a consensus decision was reached. Our inter-rater reliability, for evaluating CMBs and WMH was 0.73 and 0.85, respectively.

### Statistical Analysis

Statistical analyses were performed with SPSS version 20.0 (IBM, Chicago, IL). Continuous variables were reported as median (interquartile range [IQR]) and were analyzed with Mann–Whitney *U*-test or Kruskal–Wallis test. Categorical variables were presented as counts (proportions) and were analyzed with χ^2^-test or Fisher's exact test. Multiple comparisons were corrected by Bonferroni method. Binary logistic regression was applied to evaluate the independent relationship between antithrombotic therapy and presence of CMBs. Variables with a *P* < 0.1 in univariate analyses and potential confounders were included in multivariate analysis. When appropriate, results were reported as an odds ratio (OR) and 95% confidence interval (CI). Inter-rater variability was calculated using Cohen's kappa. Two-sided values of *P* < 0.05 were considered statistically significant.

## Results

In the study period, we enrolled 160 consecutive ischemic stroke patients with AF and/or RHD within 7 days of stroke onset at admission (West China Hospital, Sichuan University, *n* = 125; People's Hospital of Deyang City, *n* = 35). Of the included patients, there were 68 (42.5%) men and the median age at stroke onset was 71 years (age range 59–78 years). Thirty-eight patients (23.8%) had been treated with antiplatelets exclusively before admission (30 for aspirin, 4 for clopidogrel, 4 for both), 18 (11.3%) patients had been treated with warfarin exclusively, and another 10 patients switching between antiplatelets and warfarin were analyzed separately. CMBs were detected in 90 patients (56.3%). Of these, 36 had single CMBs, and 54 had multiple CMBs (≥2). CMBs were most commonly present as deep or infratentorial bleeding (53/90, 58.9%), followed by strictly lobar bleeding (37/90, 41.1%).

Table 1 shows the demographic and clinical characteristics of included patients with and without CMBs. Those patients with CMBs were more likely to have a history of hypertension (48.9 vs. 28.6%; *P* = 0.009), lower NIHSS score on admission (median 5 vs. 9; *P* = 0.001), more burden of WMH (75.6 vs. 55.7%; *P* = 0.008) and were on exclusive antiplatelet treatment three times (33.3 vs. 11.4%; *P* = 0.001) as commonly compared with those without CMBs. However, patients were comparable in terms of prior use of anticoagulants and coagulation function test.

**Table 1:** Baseline characteristics of patients with and without cerebral microbleeds.

**Characteristic**	**Total (*n* = 160)**	**With CMBs (*n* = 90)**	**Without CMBs (*n* = 70)**	***P***
Male	68 (42.5)	43 (47.8)	25 (35.7)	0.126
Age, year, median (IQR)	71 (59–78)	72.5 (63–78)	68 (56–78)	0.057
Current smoking	30 (18.8)	18 (20)	12 (17.1)	0.646
Alcohol consumption	30 (18.8)	13 (14.4)	17 (24.3)	0.114
Hypertension	64 (40)	44 (48.9)	20 (28.6)	0.009
Diabetes	39 (24.4)	21 (23.3)	18 (25.7)	0.728
Hyperlipidemia	28 (17.5)	12 (13.3)	16 (22.9)	0.116
Renal impairment	23 (14.4)	15 (16.7)	8 (11.4)	0.349
Previous stroke/TIA	39 (24.4)	24 (26.7)	15 (21.4)	0.444
NIHSS on admission, median (IQR)	7 (3–11)	5 (3–10)	9 (4–16)	0.001
PT, median (IQR)	12.1 (11.5–13.1)	12.1 (11.5–13.3)	12.2 (11.5–12.9)	0.443
APTT, median (IQR)	28.0 (24.7–30.6)	28.2 (24.5–30.9)	27.8 (25.3–30.5)	0.871
INR, median (IQR)	1.07 (1.00–1.14)	1.07 (0.99–1.14)	1.06 (1.00–1.13)	0.517
Previous use of antithrombotic agents	66 (41.3)	44 (48.9)	22 (31.4)	0.026
Exclusive use of antiplatelets	38 (23.8)	30 (33.3)	8 (11.4)	0.001
Exclusive use of aspirin	30 (18.8)	24 (26.7)	6 (8.6)	0.004
Exclusive use of anticoagulants	18 (11.3)	10 (11.1)	8 (11.4)	0.95
Presence of WMH	107 (66.9)	68 (75.6)	39 (55.7)	0.008

*CMBs, cerebral microbleeds; TIA, transient ischemic attack; NIHSS, National Institute of Health Stroke Scale; WMH, white matter hyperintensity; PT, prothrombin time; APTT, activated partial thromboplastin time; INR, international normalized ratio*.

Table 2 describes the baseline demographics and CMB distribution stratified by antiplatelet or anticoagulant use. Compared with non-antithrombotic users, those with prior antiplatelet use were older, more frequently to have CMBs, and had marginally higher prevalence of strictly lobar CMBs. Patients with warfarin had higher INR values but no significant difference was found in the presence or location of CMBs.

**Table 2:** Baseline characteristics of patients by different antithrombotic therapy.

	**No antithrombotics (*n* = 94)**	**AP therapy (*n* = 38)**	**AC therapy (*n* = 18)**	***P***
Male	41 (43.6)	17 (44.7)	6 (33.3)	0.690
Age, year, median (IQR)	70.5 (58–78)	74.5 (69–79)^*^	61.5 (51.75–71)	0.002
Current smoking	19 (20.2)	9 (23.7)	1 (5.6)	0.258
Alcohol consumption	23 (24.5)	4 (10.5)	2 (11.1)	0.143
Hypertension	34 (36.2)	21 (55.3)	4 (22.2)	0.036
Diabetes	17 (18.1)	9 (23.7)	8 (44.4)	0.055
Hyperlipidemia	12 (12.8)	6 (15.8)	6 (33.3)	0.108
Renal impairment	10 (10.6)	10 (26.3)	2(11.1)	0.077
Previous stroke/TIA	15 (16)	13 (34.2)	6 (33.3)	0.037
NIHSS on admission, median (IQR)	7 (4–13)	5.5 (2–10)	6 (2–10)	0.155
PT, median (IQR)	12.2 (11.6–13.0)	12.1 (11.4–13.3)	13.0 (12.5–14.0)^δ^	0.001
APTT, median (IQR)	27.7 (24.7–30.1)	28.2 (24.2–31.2)	29.9 (25.0–32.1)	0.141
INR, median (IQR)	1.06 (0.99–1.13)	1.07 (1.02–1.14)	1.13 (1.09–1.19)^δ^	0.001
Presence of CMBs	46 (48.9)	30 (78.9)^*^	10 (55.6)	0.007
Multi-CMBs (≥2)	26 (27.7)	17 (44.7)	7 (38.9)	0.147
Strictly lobar CMBs	18 (19.1)	14 (36.8)	4 (22.2)	0.055
Deep or infratentorial CMBs	28 (29.8)	16 (42.1)	6 (33.3)	0.397
Presence of WMH	63 (67)	28 (73.7)	7 (38.9)	0.033

*AP, antiplatelet; AC, anticoagulation; TIA, transient ischemic attack; NIHSS, National Institute of Health Stroke Scale; CMBs, cerebral microbleeds; WMH, white matter hyperintensity; PT, prothrombin time; APTT, activated partial thromboplastin time; INR, international normalized ratio*.

* *Bonferroni-adjusted P < 0.05 when AP therapy vs. no antithrombotics*.

δ *Bonferroni-adjusted P < 0.05 when AC therapy vs. no antithrombotics*.

In a multivariable logistic regression model after adjustment for age, gender, history of hypertension, history of stroke or TIA, NIHSS score, INR and presence of WMH, the exclusive use of antiplatelet agents was independently associated with the presence of CMBs (OR 3.075, 95% CI 1.175–8.045, *P* = 0.022; Table 3). In terms of CMBs' location, prior antiplatelet use was associated with strictly lobar CMBs (OR 2.635, 95% CI 1.050–6.612, *P* = 0.039; Table 3) but not with deep or infratentorial CMBs. Specifically, in a subgroup of exclusive aspirin users, the significant results remained. Prior use of aspirin was still independently related with the presence of CMBs (OR 3.095, 95% CI 1.085–8.832, *P* = 0.035) and strictly lobar CMBs (OR 3.975, 95% CI 1.506–10.495, *P* = 0.005) after controlling the above confounders. However, no significant association was found between the exclusive warfarin use and the presence or location of CMBs either in the univariate or multivariate analysis.

**Table 3:** Multivariate logistic regression analysis for the association between antithrombotic therapy and cerebral microbleeds.

	**Presence of CMBs**			**Strictly lobar CMBs**			**Deep or infratentorial CMBs**		
	**OR**	**95%CI**	***P***	**OR**	**95%CI**	***P***	**OR**	**95%CI**	***P***
Age	1.015	0.981–1.049	0.400	1.023	0.984–1.064	0.249	1.001	0.967–1.037	0.948
Male	1.668	0.800–3.481	0.173	1.281	0.566–2.900	0.553	1.489	0.717–3.091	0.285
Hypertension	1.729	0.789–3.788	0.171	1.370	0.563–3.332	0.488	1.305	0.613–2.778	0.490
History of stroke/TIA	0.896	0.375–2.144	0.806	0.856	0.319–2.297	0.758	1.001	0.423–2.370	0.997
NIHSS	0.925	0.872–0.981	0.010	0.945	0.880–1.014	0.118	0.951	0.892–1.014	0.126
INR	2.429	0.192–30.704	0.493	1.937	0.146–25.615	0.616	1.150	0.119–11.106	0.904
WMH	1.877	0.823–4.281	0.135	0.379	0.149–0.965	0.042	3.989	1.553–10.249	0.004
Exclusive use of AP	3.075	1.175–8.045	0.022	2.635	1.050–6.612	0.039	1.333	0.560–3.171	0.516
Exclusive use of AC	1.627	0.458–5.777	0.452	1.106	0.272–4.502	0.888	1.559	0.423–5.754	0.505

*AP, antiplatelet; AC, anticoagulation; TIA, transient ischemic attack; NIHSS, National Institute of Health Stroke Scale; CMBs, cerebral microbleeds; WMH, white matter hyperintensity; INR, international normalized ratio*.

## Discussion

In this study, we found that CMBs were highly prevalent in Chinese ischemic stroke patients with AF and/or RHD. Prior use of the antiplatelet agents (especially aspirin) was significantly associated with presence of CMBs and especially the strictly lobar CMBs, whereas the prior use of warfarin seemed to be not related to CMBs in any location.

The frequency of CMBs in stroke patients with AF/RHD was 56.3% in the present study, which fell broadly within the range of 18% to 68% in patients with ischemic stroke (23). When focusing on stroke patients with AF, the prevalence of CMBs was reported to be around 30% (11, 12, 24, 25). The higher prevalence of CMBs in our study might be related to ethnic differences or different imaging protocol. We carefully used the SWI sequence which has better spatial resolution and post-processing ability in detecting CMBs than conventional T2^*^-GRE sequence (18).

With the high prevalence of CMBs and a possible link to bleeding tendency, there is growing interest in demonstrating the role of CMBs in AF patients who are frequently using antithrombotic agents. Our study showed that antiplatelet use was associated with presence of CMBs in ischemic stroke patients with AF/RHD. Although controversies existed in the literature (14, 26–28), our finding is consistent with three recent meta-analyses, which all showed similar associations in patients with ischemic stroke or TIA (15, 29, 30). Some of the previous studies suggested that different type of antiplatelets may have different effects on development of CMBs. The Rotterdam Scan Study showed that aspirin was associated with lobar CMBs while clopidogrel associated with deep CMBs in general population (31, 32). A Japanese study among ICH patients reported that only aspirin, but not clopidogrel, cilostazol, or ticlopidine, was associated with the presence of CMBs (27). We also found that prior use of aspirin was associated with the presence of CMB; however, we did not evaluate the impact of clopidogrel on CMBs due to the limited number of clopidogrel users. These discrepancies among studies may be due to the difference of study population and prevalence of CMBs. Furthermore, in terms of the duration of antiplatelet therapy, a Chinese study found that CMBs are more prevalent among aspirin users with longer duration (>5 years) than those receiving aspirin <5 years (14), whereas another study from Japan failed to show this association between long duration (≥10 years) of aspirin use and CMB presence in multivariable analysis after adjusting for hypertension and other confounders (16). It is important to note that most of these studies including ours are major cross-sectional design, a conclusion on the causal relationship between antiplatelet use and development of CMBs cannot be drawn.

Different topographical associations of CMBs with the prior antiplatelet therapy were detected in our study. Prior use of antiplatelets and especially aspirin were only significantly associated with occurrence of strictly lobar CMBs rather than deep or infratentorial CMBs. Our findings are in line with the Rotterdam Scan Study (31) and the newly published meta-analysis (30). This result may be explained by the different underlying pathophysiologic mechanisms of CMBs in different locations. CMBs located in strictly lobar brain regions are associated with cerebral amyloid angiopathy, while those located in deep or infratentorial brain regions are related to hypertensive vasculopathy (5). Intriguingly, some previous study reported that the aspirin-associated hemorrhages predominantly occurred in the lobar areas (33), suggesting that aspirin favors rupture of small vessels with cerebral amyloid angiopathy. In this aspect, it is speculated that antiplatelet use would be more related to risk of lobar CMBs other than deep or infratentorial CMBs. However, due to the small sample size in subgroup analysis, there might be somewhat overfitting and possibility of chance. Moreover, whether lobar CMBs are truly attributable to CAA in patients with AF is yet to be ascertained. Further pathological studies are needed to clarify this point and explore other conditions that modulate the link between antiplatelet use and lobar CMBs.

In our study, we could not confirm an association between anticoagulation and CMBs. This result should be interpreted cautiously due to the small sample size of warfarin users. Nevertheless, previous studies have provided conflicting results regarding this question. In the Rotterdam Scan Study (31) and a Korean study of asymptomatic elderly (34), no association was found between warfarin and presence of CMBs. Another Turkish case-control study also demonstrated that warfarin treatment had no effect on CMBs in ICH patients (35). Moreover, in a longitudinal study with ischemic stroke patients on warfarin therapy for 2 years, the mean duration of warfarin treatment was not significantly related to new development of CMBs (36). Conversely, there is also evidence linking warfarin use to CMB presence. A German retrospective study of 785 patients with ischemic stroke and TIA (16.3% with AF) showed that patients with prior use of anticoagulant agents were more likely to have CMBs as compared to those without; these CMBs tended to be in lobar location (12). Unfortunately, this association was no longer present after full adjustment for demographic and clinical variables in the multivariable analysis, with the age remained the only independent factor for CMBs (12). In a large population-based study in Netherlands, higher INR values were associated with CMBs; however, we did not find the association in our study (37), which might be partly due to few patients with targeted INR. Considering INR values representing the intensity of anticoagulation that may influence the occurrence of CMBs, further prospective studies are required to clarify this association.

Notably, we also found that hypertension is an important risk factor of CMBs, which was in line with previous studies (16, 38). The chronic hypertension and associated arterial stiffness have been reported to be linked to the presence of CMBs (39) and further contribute to higher risk of hemorrhagic stroke especially after possible acute treatment such as thrombolysis (40, 41). Therefore, it is reasonable to speculate that hypertension may explained at least in part the occurrence of CMBs in addition to the effects of antithrombotic therapy. Under impaired hemostasis by antithrombotic agents, the red blood cells are prone to extravasate through the vulnerable microvasculature and this process might be aggravated by the presence of hypertension. These results suggest that vascular risk factors should be carefully monitored when prescribing antithrombotic drug treatment to patients with multiple CMBs.

Studying the relationship between CMBs, antithrombotic agents and AF/RHD is of great importance. Firstly, patients with AF have a significantly higher prevalence of CMBs and they tend to receive anticoagulation and antiplatelets more frequently than patients without AF (24). Secondly, CMBs are small blood extravasations and these asymptomatic leaks could evolve into a symptomatic ICH when hemostasis is damaged under antithrombotic therapy (42). There is now clear evidence that ICH incidence is higher in patients with CMBs than those without CMBs in antithrombotic users with AF (43), and the risk of ICH associated with CMBs is higher in Asians (10). With the aging population and growing prevalence of AF/RHD patients in China, it is essential to investigate the correlation of CMBs with antithrombotic therapy and determine how this small vessel disease marker can help analyze the benefit-risk ratio for stroke prevention strategies.

Our study has several limitations. First, this is a hospital-based study with a relatively small sample size and low number of the patients treated with oral anticoagulants. Second, subgroup analysis of different type of antiplatelets was not performed because most of our patients used aspirin. Nevertheless, the significant association of antiplatelets as whole remained in the subgroup of aspirin users. Third, the durations of drug use were not recorded due to the incomplete data in the medical records. It is possible that the durations of antithrombotic agents influence the occurrence of CMBs. Fourth, the selection bias cannot be avoided because only part of patients underwent SWI sequence due to the disease severity and cost of multiple MRI sequences. Finally, given the cross-sectional and retrospective nature of the study, it is possible that some CMBs may have occurred before use of antithrombotic agents. Therefore, further prospective investigation is required to confirm these findings.

## Conclusion

In conclusion, prior antiplatelet use is independently associated with the presence of CMBs in ischemic patients with AF and/or RHD, especially among those with lobar CMBs. Whether anticoagulants by itself could cause CMBs remains unclear. These findings warrant future prospective longitudinal studies to clarify the relationship among antithrombotic therapy, cerebral microbleed and macrohemorrhage in stroke patients with AF and/or RHD.

## Author Contributions

ML designed the study. YC, JfL, JieL, CW, and JinL collected the data. YC and JfL performed imaging analysis. YC and JfL performed statistics analysis. YC and JfL drafted the main part of the manuscript. BW and SZ provided support for imaging analysis. SZ, DW, and YW helped design the study and revised the manuscript. All authors approved the final version submitted for publication.

### Conflict of Interest Statement

The authors declare that the research was conducted in the absence of any commercial or financial relationships that could be construed as a potential conflict of interest.
